# Optimizing CMV prevention after allogenic hematopoietic stem cell transplantation: using CMV-specific cellular immunity to guide letermovir prophylaxis

**DOI:** 10.1097/BS9.0000000000000269

**Published:** 2025-12-19

**Authors:** Sisi Zhen, Xin Chen, Sizhou Feng

**Affiliations:** a State Key Laboratory of Experimental Hematology, National Clinical Research Center for Blood Diseases, Haihe Laboratory of Cell Ecosystem, Institute of Hematology and Blood Diseases Hospital, Chinese Academy of Medical Sciences and Peking Union Medical College, Tianjin, 300020, China; b Tianjin Institutes of Health Science, Tianjin, 301600, China.

We read with great interest the article by Wang et al,^1^ in which the authors used cytomegalovirus (CMV)-specific cell-mediated immunity (CMV-CMI) to guide the duration of letermovir prophylaxis. This individualized approach effectively optimized letermovir use, significantly reduced the incidence of late-onset CMV reactivation, and improved both overall survival (OS) and relapse-free survival (RFS), with a favorable safety profile.

Before letermovir became available, CMV reactivation was one of the most common complications after allogenic hematopoietic stem cell transplantation (allo-HSCT). In some patients, CMV reactivation progressed to CMV disease, increasing all-cause mortality post-transplant. With the expanding adoption of haploidentical HSCT (haplo-HSCT), this clinical challenge has become a growing concern. Furthermore, conventional preemptive anti-CMV agents, including ganciclovir and foscarnet, are associated with substantial toxicities, underscoring the need for effective CMV prophylaxis. It was against this backdrop that the introduction of letermovir emerged as a transformative advancement in CMV prevention, substantially reducing the incidence of CMV reactivation throughout the prophylactic period. However, CMV reactivation rates increased after letermovir discontinuation, even when prophylaxis was extended—a phenomenon observed in earlier studies.^2^ Recent research has primarily focused on identifying the optimal letermovir duration (eg, 100 vs 200 days). Yet, even with 200 days of prophylaxis, CMV reactivation rates remained elevated after discontinuation, suggesting that simply prolonging prophylaxis duration does not prevent viral rebound after letermovir discontinuation.^3,4^ Thus, there is an urgent clinical need for new strategies to guide letermovir prophylaxis and reduce late-onset CMV reactivation.

Protection against CMV reactivation is primarily mediated by CMV-specific T cells. After allo-HSCT, CMV reactivation often results from impaired recipient CMV-specific immunity coupled with incomplete reconstitution of donor-derived CMV-specific T cells. Our study, along with several others, has confirmed that the quantity and functionality of CMV-specific T cells can predict CMV reactivation post-transplantation.^5^ Additionally, letermovir prophylaxis may delay the immune reconstitution of CMV-specific T cells after transplantation, contributing to viral rebound after drug withdrawal.^6,7^ Therefore, using CMV-CMI to guide decisions on extended letermovir prophylaxis is well justified. This strategy has demonstrated efficacy in solid organ transplant recipients,^8,9^ but evidence in the allo-HSCT setting remains limited.

Wang et al were the first to propose CMV-CMI-guided individualized letermovir prophylaxis in allo-HSCT patients. On the one hand, comparing to a fixed-duration approach, their approach identifies patients with poor CMV-CMI recovery who would benefit most from extended prophylaxis, thereby minimizing late-onset clinically significant CMV infection (cs-CMVi). On the other hand, it avoids unnecessary prolonged prophylaxis in patients with adequate immune reconstitution. As a result, this strategy significantly reduced the incidence of late-onset cs-CMVi (9.7% vs 24.8%, *p* = 0.019) and mitigated post-LTV viral rebound, a problem commonly observed with the fixed-duration approach. Importantly, CMV breakthrough rates during prophylaxis did not increase significantly (2.8% vs 6.4%, *p* = 0.290).

The authors also comprehensively evaluated the impact of this strategy on transplant outcomes. Compared with the fixed-duration approach, the CMV-CMI-guided group showed improved 1-year OS (89.1% vs 77.1%, *p* =0.040) and RFS (88.4% vs 76.8%, *p* = 0.010). In contrast, simply extending letermovir prophylaxis in other studies did not significantly improve transplant outcomes^3^. underscoring the advantage of the CMV-CMI-guided strategy. Safety analyses confirmed that the CMV-CMI-guided approach did not increase the risk of other viral infections, such as Epstein-Barr virus reactivation or post-transplant lymphoproliferative disorder.

Furthermore, the authors performed risk stratification based on clinical factors and evaluated the effect of CMV-CMI guidance in different risk groups. They found that this strategy did not affect late-onset CMV reactivation in low-risk patients but significantly reduced it in high-risk patients. These results indicate that CMV-CMI-guided LTV prophylaxis is particularly beneficial for high-risk individuals, helping to define its target population.

Some limitations should be noted. The study was nonrandomized, which may affect the generalizability of the findings. The method for assessing CMV-specific T-cell function also requires further refinement. Larger studies are needed to determine the optimal CMV-CMI cutoff and to validate it externally. Although interferon-gamma (IFN-γ) is a major cytokine produced by CMV-specific T cells, other cytokines such as interleukin-2, tumor necrosis factor-alpha, and chemokine C-C motif ligands 4 may also contribute to antiviral immunity. Therefore, relying solely on IFN-γ may not fully capture the risk of CMV infection. Finally, the cellular source of IFN-γ (CD4^+^ vs CD8^+^ T cells) was not specified.

In summary, this study demonstrates that a CMV-CMI-guided strategy reduces late-onset CMV reactivation following the discontinuation of prophylaxis in allo-HSCT recipients, particularly in high-risk patients, and leads to significantly better OS and RFS.
